# Adherence to the Qatar dietary guidelines: a cross-sectional study of the gaps, determinants and association with cardiometabolic risk amongst adults

**DOI:** 10.1186/s12889-018-5400-2

**Published:** 2018-04-16

**Authors:** Mohammed Al Thani, Al Anoud Al Thani, Walaa Al-Chetachi, Badria Al Malki, Shamseldin A. H. Khalifa, Ahmad Haj Bakri, Nahla Hwalla, Farah Naja, Lara Nasreddine

**Affiliations:** 1 Public Health Department, Ministry of Public Health, Doha, Al Rumaila West, Doha, Qatar; 2 Health Promotion and Non Communicable Disease Prevention Division, Ministry of Public Health, Doha, Al Rumaila West, Doha, Qatar; 3 Nutrition and Food Sciences Department, Faculty of Agriculture and Food Sciences, American University of Beirut, P.O. BOX 11-0.236 Riad El Solh, Beirut, 11072020 Lebanon

**Keywords:** Adherence, Dietary guidelines, Adults, Cardiometabolic risk, Qatar

## Abstract

**Background:**

The Qatar Dietary Guidelines (QDGs) were developed as part of the national strategy to prevent chronic diseases. This study aims at characterizing gaps between the QDGs and usual dietary and lifestyle patterns in Qatar, identifying demographic and socioeconomic determinants of adherence to the QDGs and investigating the association between adherence and cardiometabolic risk.

**Methods:**

This study is based on the Qatar National STEPwise cross-sectional survey which was conducted on a nationally representative sample of Qatari adults, aged 18 to 64 years (*n* = 1109). Data collection included socio-demographic characteristics, lifestyle factors, anthropometric (weight, height and waist circumference (WC)), and blood pressure measurements. The dietary intake of participants was evaluated using a non-quantitative food frequency questionnaire (FFQ). Biochemical assessment was performed to measure the fasting levels of blood sugar, triglycerides (TG) and HDL cholesterol. The metabolic syndrome (MetS) was defined as the presence of three or more cardiometabolic risk factors. To examine adherence to the guidelines, each specifc recommendation was matched to corresponding data drawn from the survey. To investigate the association of sociodemographic, lifestyle and cardiometabolic characteristics with adherence to the QDGs, an adherence score was calculated.

**Results:**

More than 83% of adults did not meet the recommendations for vegetables, fruits, whole grains, legumes and high fibre intakes, 70% were overweight or obese, 50–72% reported frequent consumption of sweetened beverages and sweets, and 47% reported frequent consumption of fast foods. Younger adults, the unemployed, the least educated and those not married had lower adherence to the QDGs. Adherence was inversely associated with elevated WC (OR: 0.88, 95% CI:0.82–0.95) and the MetS (OR:0.84,95% CI:0.74–0.96).

**Conclusions:**

Building on the identified gaps and vulnerable population groups, the study findings should provide a road map for the prioritization of interventions and the development of culture- specific programs aiming at promoting adherence to dietary guidelines in Qatar, while serving as a model to other countries in the region.

## Background

The Middle-East and North Africa (MENA) region, and particularly countries of the Gulf Cooperation Council (GCC), have witnessed over the past three decades, significant social, economic and demographic changes that have greatly influenced the nature and magnitude of health problems and the burden of associated morbidity [1]. Qatar, which has recently emerged as the richest country in the world in terms of Gross Domestic Product (GDP) per capita, is no exception to this trend [2]. Among other changes, Qatar has experienced fast rates of development, mechanization, and urbanization, with concurrent shifts in diet, physical activity and body composition [3, 4]. These shifts and changes are implicated in the nutrition transition phenomenon, which is characterized by an increase in the intakes of energy, animal fat, added sugars, and salty foods [3, 5]. Through these distinctive changes in food consumption patterns, the nutrition transition has been linked to the escalating burden of non-communicable diseases (NCDs) in Qatar, which contribute 69% of mortality in the country [3, 4, 6, 7]. In 2011, Qatar’s expenditure on healthcare was estimated at 3.32 billion $US, which represent a 27% increase compared to 2010 [8].

As part of its National Health Strategy [9] and Nutrition and Physical Activity Plan to ‘reduce morbidity and mortality attributable to chronic non-communicable diseases’ [10], Qatar developed its first national dietary guidelines in 2014 [11]. The development of the Qatar Dietary Guidelines is in line with the recommendations of the Food and Agriculture Organization of the United Nations (FAO)/World Health Organization (WHO) International Conference on Nutrition (1992), which has called for national, culture-specific dietary guidelines to foster and instill healthy eating habits and lifestyles in the general population [12, 13]. The development of the Qatar Dietary Guidelines (QDGs) was led by the Ministry of Public Health (formally known as Supreme Council of Health), Health Promotion and Non-communicable Diseases, in collaboration with the National Dietary Guidelines Task Force, which included nutritionists, and representatives from various stakeholders and institutions across Qatar, such as Qatar National Food Security Program, academic and research institutions, and medical associations [11, 14, 15].

Beyond development, the implementation of these guidelines is a complex task that requires enabling factors at both the personal and environmental levels [16]. In today’s complex societies, a “one size fits all” communication and implementation approach will probably be ineffective in instilling behavioral change [16]. As such, the promotion and implementation of dietary guidelines necessitate evidence-based information on the existing gaps between the guidelines and the dietary and lifestyle practices of the population, and on the demographic and socioeconomic factors affecting adherence to these guidelines within the country [16]. Furthermore, the effective implementation of dietary guidelines in any given population would necessitate not only the measurement of adherence itself, but also knowledge of whether adherence to these guidelines could reduce the risk of chronic diseases [16, 17]. Even though efforts were made by some Arab Middle-Eastern countries to develop national dietary guidelines [13], there have been no studies or investigations that went beyond the development aspect, to characterize the population’s adherence to the developed guidelines, identify barriers and potential factors linked with poor adherence and examine the effects of adherence on the population’s health profile.

Based on data provided by the Qatar National STEPwise Survey (2012) [18], this paper aims at 1) characterizing the gaps between the QDGs and usual food consumption and lifestyle patterns in Qatar; 2) identifying demographic and socioeconomic determinants of adherence to the QDGs; and 3) investigating the association between adherence to the QDGs and cardiometabolic risk amongst Qatari adults.

## Methods

Data from the Qatar National STEPwise Survey (2012) was used in order to examine adherence to the Qatar Dietary Guidelines, the gaps, determinants and association with cardio-metabolic risk factors,.

The study focuses specifically on the four dietary and lifestyle-related guidelines of the QDGs, which represent the founding elements of the “Qatar National Nutrition and Physical Activity Plan 2011-2016”, and which include “Eat Healthy Choices from the 6 Food Groups”, “Maintain a Healthy Weight”, “Limit Sugar, Salt and Fat” and “Be Physically Active”.

### Methods used in the Qatar national STEPwise survey

The survey was carried on a nationally representative sample of Qatari adults (18–64 years old) and followed the guidelines set by the WHO for the STEPwise surveys of NCD risk factors [19]. Details describing the Qatar National STEPwise Survey have been described elsewhere [20]. In brief, the selection of the households (primary sampling units) was random using multi-stage cluster sampling. The selection of the cluster was based on probability proportional to size sampling and the cluster consisted of 60–70 adjacent blocks. In each of the 7 municipalities making up the state of Qatar, 95 clusters were selected, within each 30 households were identified randomly [21]. Eligibility criteria included 1) Qatari nationality and 2) 18 to 64 years of age. In total, 2850 households were approached, and 2496 agreed to participate in the study (response rate = 88%).

For the purpose of this study, data pertinent to male and female participants with no known diagnosis of hypertension, diabetes, dyslipidemia or any other health condition that may affect their dietary intakes were included (*n* = 1539). Subjects were excluded from data analyses if they had missing information on adherence to QDGs or if they were diagnosed or were receiving treatment for hypertension, diabetes, dyslipidemia or any other health condition that may have an impact on their dietary intakes. Out of a total of 1539, 1109 subjects had no missing data, 377 had one and 53 had 2 or more missing data. Hence the total number of participants whose data was included in the analysis for this study was *n* = 1109.

During the survey, data collection followed the three steps of the WHO STEPwise approach, which included a multicomponent questionnaire examining socio- demographic, dietary and lifestyle factors, anthropometric measurements, as well as biochemical assessment. Data collection was carried out by trained nutritionists through one-to-one interviews with the participants, in 2012.

Through the questions of the multicomponent questionnaire, the following participants’ characteristics were examined: age, sex, marital status, education level, employment status, parental consanguinity (parents being first cousins), family history of diabetes and hypertension, number of meals not prepared at home, use of vegetable oils or animal fats in cooking, smoking and physical activity. The Arabic version of the WHO physical activity standard questionnaire (Arabic version) was used to assess the frequency, duration and intensity of each activity amongst the study participants [19]. In order to examine the dietary intake of survey participants, a non-quantitative food frequency questionnaire (FFQ) was used. The food list of this FFQ consisted of thirteen food groups: refined grains, whole grains, fruits, vegetables, beans, milk and dairy products, meat, poultry, fish and sea food, sweets, sweetened beverages, fast foods, and natural juices. The frequency of consumption of each of the food groups was determined based on the number of days per week during which the food group was consumed. In order to enhance the clarity of the FFQ and aid subjects to match their food consumption to the food groups listed in the questionnaire, field workers were trained to provide examples of foods belonging to each food group, using show cards for illustrations. As a non-quantitative FFQ, the questionnaire did not contain questions regarding the serving size consumed from each food group, except for food groups such as fruits and vegetables whereby the number of serving consumed per day was assessed. Although they do not provide quantification of dietary intake, the non-quantitative FFQs are considered valuable assessment tools that provide an overview of dietary habits and trends in population-based studies [22]. In order to improve the clarity and culture sensitivity of the dietary intake assessment, pilot testing of the FFQ was carried out on a sample of Qatari adults prior to the initiation of data collection. The pilot test of the FFQ resulted in inclusion of specific examples of foods pertinent to the Qatari context. For instance, Ka’ak (dry form of bread) commonly consumed with tea was specified under refined grains. Similarly, dates, an integral part of the Qatari diet, were provided as example of the ‘fruits’ food group. The data collection for physical activity and dietary intake (FFQ) referred to a typical week of the survey participants. The reference duration for recall was the past year. The multicomponent questionnaire also included questions about dietary practices such as the number of meals consumed outside the home and the utilization of vegetable oils vs. animal fats in cooking.

Blood pressure (mmHg) and anthropometric measurements consisted of the following: height (cm), weight (kg), and waist circumference (WC) (cm). An Omron sphygmomanometer (Omron BP785; Shanghai, China) was used to measure blood pressure. Three readings within five minutes- intervals were obtained for systolic and diastolic blood pressure and the average of the second and the third readings were calculated and used in the study. Weight, height and WC were measured using standardized techniques and calibrated equipment and as part the quality assurance protocol, all anthropometric measurements were taken twice [23, 24]. For differences exceeding 0.5 cm for height, 0.1 kg for weight and 1 cm for WC, a third measurement was taken [25]. The average of the two closest values was calculated and adopted in the study. Waist circumference measurements were taken at midpoint between the lower margin of the last palpable rib and the top of the iliac crest, using a stretch-resistant tape which was kept horizontal and parallel to the ground [23]. The measurement was done after the subject’s normal expiration, with the subject standing “with arms at the sides, feet positioned close together, and weight evenly distributed across the feet” [23].

The biochemical measurements were conducted using dry chemistry equipment and supplies and included fasting blood glucose (mg/dl), triglyceride (TG) (mg/dl) and high-density lipoprotein (HDL) (mg/dl). The blood samples were obtained after survey participants had a minimum of 12 h fast. Details about the methods used to analyze the blood samples are described elsewhere [26].

Cardiometabolic risk factors were assessed based on the harmonized definition of the metabolic syndrome [27], as follows: Blood pressure greater or equal to 130/85 mmHg, a fasting TG level greater or equal to 150 mg/dl, a low fasting HDL cholesterol level (< 40 mg/dl males, < 50 mg/dl females) and an elevated fasting blood sugar ≥100 mg/dl [27]. Elevated WC was assessed based on the Middle-Eastern cutoffs of ≥94 cm in men and ≥ 80 cm in women [27]. The metabolic syndrome (MetS) was defined as the presence of three or more of the aforementioned cardiometabolic risk factors [27].

### Ethics, consent and permissions

Ethical approval was obtained from the Ministry of Public Health and the Ministry of Development Planning and Statistics, Doha, Qatar. All subjects provided informed consent for their participation.

### Assessment of adherence to the Qatar dietary guidelines

The QDGs consisted of eight guidelines of which 4 targeted dietary intake, body weight and physical activity while the remaining 4 guidelines addressed water consumption, safe and clean food and preparation, protection of the environment, as well as taking care of the family. Within each of the guidelines, several messages were depicted to simplify and operationalize the guideline. In order to examine adherence to the Qatari guidelines related to dietary intake, body weight and physical activity, each message within these guidelines was matched to corresponding data source (variable) drawn from the WHO STEPwise survey (Table 1). Two approaches were used to assess adherence to recommendations:

1. **If the recommendation included a reference cut off**, then this cut off was used to determine adherence. For instance, for the following guideline “Consume ≥ 3 servings of vegetables per day”, the cutoff was set at 3, with subjects consuming less than 3 being classified as not adherent while those consuming 3 or more classified as adherent.
2. **If the recommendation did not include a reference cutoff and rather included a comparative statement (less, avoid, more, favor, substitute)**, then the distribution of the sample was divided into quartiles and the reference was considered as the cut off for either the first or fourth quartile, depending on whether the guideline included ‘less’ or ‘more’ consumption. For instance, for the following guideline ‘Consume sweets less often (days per week)’, the first quartile of the population distribution for sweets consumption corresponded to 2, hence the cut off for the guideline was set at 2.

**Table 1:** List of the Qatar Dietary Guidelines and corresponding criteria stemming from WHO STEPwise survey (*n* = 1109)

Qatar Dietary Guidelines	Criteria used to assess adherence
Aim for 3–5 servings of a variety of vegetables every day.	Consume ≥3 servings of vegetables per day
Aim for 2–4 servings of a variety of fruit every day.	Consume ≥2 servings of fruits per day
Favor whole fruit over juices.	Consume whole fruits more often than natural juices (days per week)
Substitute refined grains with whole grain breads and cereals.	Consume whole grains more often than refined grains (days per week)
Eat legumes daily.	Consume beans daily (7 days per week)
Maintain a daily consumption of skimmed or low fat milk and dairy products.	Consume dairy products daily (7 days per week)
Eat a variety of fish at least twice a week.	Consume fish at least twice per week (days per week)
Eat less fast foods and processed foods.	Consume fast food less often (<first quartile-2 days/week)
Limit sweetened foods ^a^	Consume sweets less often (<first quartile-2 days/week)
Avoid sweetened beverages ^a^	Consume sweetened beverages less often (<first quartile-2 days/week)
Use healthy vegetable oils such as olive, corn	Use vegetable oil instead of animal fat in cooking
Eat home-made food more often	Consume meals outside home less often (<first quartile-3 meals per week)
Eat more high fiber foods.	Consume high fiber foods more often (Fruits, vegetables, wholegrain, and legumes)-times per week (>4th quartile-21 times per week)
Maintain a healthy weight	Maintain a healthy body mass index (Normal body mass index (BMI)^b^
Spend less time sitting	Sit less often (first quartile-240 min per day) -minutes sitting per day
Adults should do moderate aerobic physical activity at least 5 days per week (for at least 30 min) and/or vigorous intensity aerobic physical activity at least 3 days per week (for at least 20 min) ^c^	Do at least 150 min per week of moderate physical activity or at least 60 min per week of vigorous physical activity (minutes per week)
Overall adherence score (calculated as the sum of the number of recommendations the study participants adhered to)	The score ranged between 0 and 16.

^a^Sweetened foods and beverages include all foods and drinks with added sugars such as sucrose or high fructose corn syrup. Examples include soft drinks, sodas, sweetened juices, sweetened Tea/coffee, cakes, deserts, chocolate, jams, etc.

^b^Normal BMI defined as BMI ranging between 18.5 and 24.9 kg/m^2^

^c^Vigorous-intensity activity is defined as activity that needs large amounts of effort and causes “large increases in breathing or heart rate”, such as running, carrying or lifting heavy loads and construction work; moderate-intensity activity, however, is defined as activity that needs moderate amounts of effort and causes “small increases in breathing or heart rate”, such as brisk walking, housework or carrying light loads [19]

It remains important to note that no data was available to assess adherence to guidelines related to water intake, safe and clean food preparation, protecting the environment and taking care of the family. A total adherence score was computed as a sum of the number of recommendations the study participants adhered to. The adherence score had a maximum of 16. (Table 1).

### Statistical analyses

The distribution of the study sample was compared to that of the Qatari population, in terms of age, sex and geographical area (Appendix) [28]. Given the considerable differences between these distributions, sampling weights were applied in order to correct for the selection probabilities. The weights were calculated as the inverse of the sampling fractions. Adherence to each of the messages of the QDGs was expressed as n (%) in the total study sample as well as among males and females. Chi-square test was used to examine differences in adherence between males and females. Descriptive statistics of the adherence score were then calculated for the total study sample and across socio-demographic and lifestyle factors and comparisons were made using the independent t-test and ANOVA. The associations between the socio-demographic and lifestyle factors with adherence were further examined using multiple linear regression analyses, whereby the adherence score was the dependent variable, and the sociodemographic and lifestyle factors were the independent variables. Multiple logistic regression models were used to evaluate the associations between adherence to the QDGs and cardiometabolic risk factors, including elevated WC, elevated blood pressure, hyperglycemia, low HDL, hypertriglyceridemia and MetS. In these models the independent variable was adherence to the QDGs and the dependent variables were each of the cardiometabolic risk factor listed earlier. The logistic regression models were adjusted for sociodemographic and lifestyle variables that were found to be significantly associated with adherence in the simple linear regression analysis and included, in addition to age and sex, education, marital status, and employment status. All analyses were two tailed and a *p*-value < 0.05 was considered statistically significant. The Statistical Package for the Social Sciences (SPSS; version 14.1) was used for all computations [29].

## Results

Descriptive characteristics of the study population are presented in Table 2. The study population was evenly distributed between males (51%) and females (49%). Almost 38% of study participants had a university or higher education level and 68% were married. Only 38% were not working and approximately 18% were smokers at the time of the interview (Table 2).

**Table 2:** Socio-demographic and lifestyle characteristics among Qatari adults (n = 1109)

	n = 1109	
	n	%
Sex		
Male	570	51.4
Female	539	48.6
Age (years)		
18–24	264	23.8
25–34	314	28.3
35–44	332	29.9
45–54	162	14.6
55–64	37	3.3
Education		
Up to high school level^a^	688	62.1
University/graduate level	420	37.9
Marital Status		
Not married	355	32.0
Married	754	68.0
Employment status		
Not working (including housewife)	417	37.6
Working	691	62.4
Parental Consanguinity		
No	679	61.2
Yes	430	38.8
Family history of diabetes		
No	402	36.2
Yes	707	63.8
Family history of High blood pressure		
No	421	38.0
Yes	688	62.0
Smoking Status		
Nonsmoker or past smoker	914	82.4
Current smoker	195	17.6

^a^This category includes: no schooling, elementary, intermediate schooling and high school

Mean dietary intakes, physical activity level and BMI of the study population are shown in Table 3. The average intakes of vegetables and fruits were estimated at 0.89 ± 1.09 and 1.44 ± 1.44 servings /day, respectively. The average frequencies of consumption of whole grains, beans, and fish were below two days per week, while that of sweets approached four per week. Mean BMI was of 28.83 ± 6.76 Kg/m^2^ and the average sitting time was estimated at 189.99 ± 192.19 min/day (Table 3).

**Table 3:** Mean^a^ dietary intakes, physical activity level and BMI of the study population

	Mean ± S D
Servings of vegetables per day	1.44 ± 1.44
Servings of fruits per day	0.89 ± 1.09
Intake of whole grains (days/week)	1.89 ± 2.63
Intake of beans (days/week)	1.65 ± 1.58
Intake of dairy (days/week)	5.84 ± 1.97
Intake of fish (days/week)	1.96 ± 1.57
Intake of fast food (days/week)	1.86 ± 2.00
Intake of sweets (days/week)	3.72 ± 2.67
Intake of sweetened beverages (days/week)	2.37 ± 2.61
Intake of meals outside home (meals per week)	2.53 ± 2.40
Intake of high fiber foods (times/week)	12.98 ± 5.54
Body Mass Index (Kg/m^2^)	28.83 ± 6.76
Minutes sitting per day	189.99 ± 192.19
Moderate physical activity (minutes/week)	705.19 ± 974.65
Vigorous physical activity (minutes/week)	569.96 ± 742.85

^a^Values in this table represent weighted means and their standard deviations

The proportions of participants adhering to the various recommendations of the QDGs for the overall study population and by gender are presented in Table 4. The lowest adherence proportion was noted for beans, vegetables and fiber intakes, with only 4–11% of the study population meeting the recommendation for these foods groups. Similarly, less than 17% of study participants met the recommendations for fruits or whole grains intake. Almost 70% (69.7–71.4%) of participants adhered to recommendations regarding dairy products and sitting less often. While the majority of study participants used vegetable oil in lieu of animal fat in cooking and reported a less frequent consumption of meals outside home, only 50% (50.2–54.6%) were consuming fish, fast foods, and sweetened beverages according to recommendations. Maintaining a healthy body was only observed among 26.8% of participants, with 70% being either overweight or obese and 3% underweight (data not shown). Compared to females, males were more likely to consume fruits, fish, sweets, vegetable oil instead of animal fat and follow a physical activity level according to recommendations (Table 4). On the other hand, higher proportions of female participants adhered to the recommendations related to dairy products and consuming meals outside the house.

**Table 4:** Weighted proportions of Qatari adults (n, %) adhering to recommendations of the Qatar Dietary Guidelines

Criteria used to assess adherence	Total *n* = 1098	Males *n* = 565	Females *n* = 533	p-value^b^
Consumption of ≥3 servings of vegetables per day	121 (11)	59 (10.4)	62 (11.6)	0.53
Consumption of ≥2 servings of fruits per day	181 (16.5)	107 (18.9)	74 (13.9)	0.03*
Intake of whole fruits >natural juices (days/week)	372 (33.9)	187 (33.1)	185 (34.7)	0.57
Intake of whole grains > refined grains	162 (14.7)	86 (15.2)	76 (14.3)	0.66
Daily intake of beans	48 (4.4)	19 (3.4)	29 (5.4)	0.09
Daily intake of dairy products	765 (69.7)	358 (63.4)	407 (76.4)	0.000**
Intake of fish ≥ twice/week	599 (54.6)	344 (60.9)	255 (47.8)	0.000**
Intake of fast food <2^c^ days/week	583 (53.1)	287 (50.8)	296 (55.5)	0.12
Intake of sweets <2^c^ days/week	303 (27.6)	190 (33.6)	113 (21.2)	0.000**
Intake of sweetened beverages <2^c^ days/week	551 (50.2)	272 (48.1)	279 (52.3)	0.16
Use of vegetable oil instead of animal fat	1068 (97.2)	557 (98.4)	511 (95.9)	0.01*
Meals outside home <3^c^ meals/week	811 (73.9)	399 (70.6)	412 (77.3)	0.01*
Intake of high fiber foods ≥21^d^ times/week	114 (10.4)	61 (10.8)	53 (9.9)	0.64
Normal Body Mass Index	295 (26.8)	144 (25.4)	151 (28.3)	0.28
Sitting <240^c^ minutes /day	784 (71.4)	410 (72.6)	374 (70.2)	0.38
≥150 min/week of medium aerobic activity or 60 min/week of high aerobic activity	858 (78.1)	482 (85.3)	376 (70.5)	0.000**

Values in this table represent n (%)

^b^Significance was derived from chi-square test. *p < 0.05; **p < 0.01

^c^Belongs to the 1st quartile

^d^Belongs to the 4th quartile

Table 5 describes the association of the sociodemographic and lifestyle factors with adherence to QDGs, as assessed by the adherence score. Using chi square test, age, education level, marital status, and employment status were associated with adherence. The results of the multiple linear regression showed that participants aged 35 years or older had significantly higher adherences scores. Furthermore, a university or graduate levels of education level were associated with a greater adherence compared to high school levels (B = 0.27, 95% CI: 0.02; 0.51). Married participants, compared to single subjects, had higher adherence score (B = 0.83, 95% CI: 0.54; 1.12). Employment status was also found to be associated with adherence, whereby employed individuals had higher adherence scores (B = 0.48, 95% CI: 0.19;0.77) (Table 5).

**Table 5:** Weighted association of socio-demographic and lifestyle characteristics with QDG adherence score among Qatari adults

	Adherence score		*P*-value^a^	B	95% CI ^b^
	mean	SD			
Socio-demographic characteristics					
Sex					
Male	7.01	2.04	0.22	–	–
Female	6.85	2.04		0.05	−0.22;0.32
Age (years)					
18–24	5.80	1.67^†^	0.000**	–	–
25–34	6.54	2.05^‡^		−0.02	−0.40;0.36
35–44	7.59	1.82^§^		0.90	0.48;1.32**
45–54	8.03	1.93^§^		1.44	0.97;1.91**
55–64	7.79	1.75^§|^		1.41	0.78;2.05**
Education					
Up to high school level^c^	6.63	1.93	0.000**	–	
University/graduate level	7.34	2.12		0.27	0.02;0.51*
Marital Status					
Not married	5.99	1.92	0.000**	–	**–**
Married	7.32	1.96		0.83	0.54;1.12**
Employment status					
Not working (including housewife)	6.57	1.92	0.000**	–	–
Working	7.12	2.08		0.48	0.19;0.77**
Parental Consanguinity					
No	6.98	2.11	0.35	–	
Yes	6.86	1.94		0.08	−0.15;0.31
Family history of diabetes					
No	6.80	1.96	0.09	–	–
Yes	7.01	2.09		0.12	−0.14;0.37
Family history of High blood pressure					
No	6.84	2.01	0.23	–	–
Yes	6.99	2.06		0.07	−0.18;0.33
Lifestyle characteristics					
Smoking Status					
Nonsmoker or past smoker	6.97	2.03	0.23	–	–
Current smoker	6.77	2.10		−0.26	−0.59;0.07

^*^p < 0.05; ^**^ *p* <0.001

B: unstandardized regression coefficient, and their 95% confidence intervals

^a^Significance is derived from Chi-square test

^b^Results of the multiple linear regression

^c^This category includes: no schooling, elementary, intermediate schooling and high school

^†‡§||^Different symbols superscripts indicate significantly different numbers (Tukey post-hoc test)

Table 6 displays the association between adherence to the QDGs, as assessed by the adherence score, and each of the cardiometabolic risk factors considered in this study. Adherence was significantly inversely associated with elevated WC and MetS. The results showed that one unit increase in the adherence score led to 12% and 16% decrease in the odds of elevated WC and MetS respectively, with corresponding odds ratio of OR:0.88, 95% CI:0.82–0.95 and OR:0.84, 95% CI:0.74–0.96 (Table 6).

**Table 6:** Association ^a^ between adherence score, metabolic syndrome and its abnormalities among Qatari adults

	n(%)	Adjusted OR	95% CI	*P*-value
Elevated WC^b^	616(60.1)	**0.88**	**0.82;0.95**	0.000**
Elevated blood pressure^c^	209(20.0)	0.97	0.89;1.05	0.41
Hyperglycemia^d^	82(14.4)	0.89	0.78;1.01	0.07
Low HDL^e^	224(38.3)	0.98	0.90;1.08	0.69
High TG^f^	68(12.1)	0.93	0.81;1.07	0.30
Metabolic syndrome ^g^	86(16.3)	**0.84**	**0.74;0.96**	0.01*

^*^p < 0.05; ^**^ *p* <0.001

Abbreviations: *WC* waist circumference, *HDL* High-density lipoprotein, *TG* triglyceride

^a^Associations examined by multiple logistic regression. Each regression model was controlled for age, sex, marital status, education and employment status (sampling weights were applied)

^b^Elevated WC (≥94in males, ≥80 cm in females); Reference group: normal WC: < 94 cm in males, < 80 cm in females

^c^Elevated blood pressure (≥130SBP or ≥ 85 DBP); Reference group: normal BP: < 130 SBP and < 85 DBP

^d^Hyperglycemia (≥100 mg/dl); Reference group: blood glucose < 100 mg/dl

^e^Low HDL (< 40 mg/dl in males, < 50 mg/dl in females); Reference group: HDL ≥ 40 mg/dl in males, ≥50 mg/dl in females

^f^High TG (≥150 mg/dl); Reference group: TG < 150 mg/dl

^g^Metabolic syndrome is defined as having three or more of the following abnormalities: elevated WC, elevated blood pressure, hyperglycemia, low HDL and high TG

## Discussion

The present study has identified the main gaps between the QDGs and the dietary and lifestyle practices of Qatari adults, documented a substantial shortfall as to the proportion of adults adhering to the QDGs, and outlined the priority areas for intervention to foster adherence to these guidelines. The study has also characterized the vulnerable population groups that should be targeted by specific dietary and lifestyle-based interventions and confirmed the cardiometabolic benefits of adherence to the QDGs.

The greatest gaps were observed for the intakes of fruits, vegetables, whole grains and legumes, and consequently high fibre foods, whereby more than 83% of Qatari adults did not meet the QDG recommendations for these food groups. This is in accordance with findings reported by several other studies conducted in other parts of the world [30–32]. The observed gaps in adherence are of concern given that low intakes of these food groups have been linked with adverse health outcomes and higher risk of NCDs [33]. For instance, in their estimation of cardiometabolic disease (CMD) mortality “attributable to dietary and metabolic risk factors” in the Middle-East and North Africa (MENA) region, Afshin et al. (2015) showed that low intakes of fruits and whole grains were the main dietary risk factors for mortality in the region, representing 12% and 11% of CMD deaths, respectively [33]. Adequate consumption of fruits, vegetables, whole grains and legumes promote higher intakes of dietary fibre, which, through its colonic, intrinsic and hormonal effects has been associated with increased satiety, increased fat oxidation, decreased postprandial glycaemia and increased insulin sensitivity, all of which may contribute to the prevention of NCDs [34]. Taken together, these findings indicate that one of the main priorities areas to be targeted by nutrition interventions in Qatar is promoting adherence to the QDG recommendations on fruits, vegetables, and other high fibre foods. This would represent a cost-effective approach to improve the health profile of the population in Qatar, a country that is currently plagued by one of the highest burdens of NCDs in the world. The fact that a high proportion of Qatari adults reported frequent consumption of sweetened beverages and sweets (50–70%), with almost half consuming inadequate levels of fish, further highlights the population’s increased risk for NCDs [33] and their poor alignment with the recommendations of the QDGs.

The results of this study have also documented a large adherence gap as to the proportion of individuals maintaining a healthy body weight, with 70% of Qatari adults being overweight or obese. These prevalence rates, which are amongst the highest in the world [3], do not bode well for the health and well-being of the Qatari population, given the well-established association between adiposity and chronic disease risk [35]. As such, the study findings highlight the prevention of obesity as another priority area for intervention in Qatar, where complementary strategies targeting diet, physical activity and sedentarity ought to be implemented.

The present study identified specific demographic and socioeconomic factors associated with adherence to the QDGs. Even though overall adherence to the QDGs did not differ between males and females, gender disparities in adherence to specific recommendations within the QDGs were observed. This was particularly true for physical activity, whereby men were found to be significantly more active than women. Similar gender disparities were described by Mabry et al. (2009) in a review of physical activity in countries of the GCC, highlighting the unique socio-cultural and environmental factors modulating the lifestyles of men and women in this region [36]. Gender-specific disparities in adherence to specific dietary recommendations were also observed in this study, whereby, compared to women, Qatari men were more likely to consume fruits, fish, sweets and vegetable oil according to recommendations. This is in disagreement with studies conducted in other parts of the world, reporting women as being more health-conscious and followers of dietary recommendations than men [37–39]. These findings warrant further investigations of the social and cultural factors modulating the dietary habits of men and women in Qatar, and possibly in other countries of the GCC.

In this study, age, employment status and higher educational levels were identified as characteristics associated with increased adherence to the QDGs. In agreement with our findings, several other studies suggested that increasing age was associated with higher intakes of fruits and vegetables [40, 41] and with overall healthier diets [41, 42]. The observed age gradient in adherence to the guidelines may reflect the state of nutrition transition, from traditional dietary habits to westernized dietary patterns, a phenomenon that typically manifests itself in younger age groups as is currently experienced by many countries of the Eastern Mediterranean region [37, 39, 43]. The observed association with employment status and higher educational levels suggest a possible socioeconomic gradient in healthy eating, which is in line with findings reported by several previous studies [44, 45]. Marital status was also found to be associated with adherence scores in this study, with married individuals having higher adherence with the QDGs. This may be a reflection of the culture-specificity of Qatar and countries of the region, whereby married individuals tend to more frequently consume home-cooked meals within the domestic setting.

Findings of the present study showed that higher adherence to the QDGs amongst Qatari adults was associated with lower odds of MetS and elevated WC. The QDGs aim at instilling healthier lifestyles, promoting physical activity and the consumption of diets rich in fruit, vegetables, legumes, whole grain, and fish. Substantial evidence suggests that adherence to such dietary patterns could decrease the risk of all-cause mortality, obesity, NCDs and metabolic abnormalities [33, 46, 47]. The higher content of polyphenols, antioxidants, fibre and polyunsaturated fats may work in concert to decrease oxidative stress, temper the inflammatory response, increase insulin sensitivity, improve postprandial glycemic control and limit postprandial lipid excursions, all of which may contribute to the protective effects of these diets against cardiometabolic risk [34, 48–50] . Physical activity and decreased sedentary behavior, which are also included in the QDGs, have been shown to prevent the onset of chronic diseases and to decrease the risk of developing the MetS, independent of aerobic fitness and obesity [51, 52]. The observed association between overall adherence to the QDGs and decreased risk of the MetS risk may be viewed as an evidence-based confirmation of the health promoting potential of these guidelines, a fact that should be built upon and highlighted in the implementation of the QDGs at the national level.

The results of this study should be considered in light of the following limitations. First, the findings of this study could not infer causality given its cross-sectional design. However, in order to decrease possible reverse causation, subjects who reported diagnosis with a chronic disease that may affect their dietary habits were excluded from the study. This was deemed necessary given that information on whether subjects were following therapeutic diets or had received dietary consultations was not collected in this study. Second, dietary intake and physical activity assessment were self-reported and subject to recall error. Such an error would result in attenuation of the observed associations between exposure and MetS. It remains important to note that the FFQ and the physical activity assessment tools used in this study were not validated and were rather adapted from the WHO STEPs questionnaire. The use of this questionnaire in previous studies amongst Qataris has however led to plausible results [20, 53]. Third, as observed in most questionnaire-based surveys, the interview approach may incur a social desirability bias: the participating subjects may have responded in a way that they perceived as more favorable or acceptable to the interviewer [54]. For instance, this may explain the observed high proportion of subjects adhering to the physical activity recommendations (78%). The study population included those subjects that were not previously diagnosed with chronic disease and were therefore more likely to engage in physical activity, but it remains important to acknowledge that the high proportion of subjects adhering to the recommendations may be related to the self-reported nature of physical activity assesment. Previous studies have shown that self-reported adherence estimates were considerably higher than those measured using more objective methods of physical activity assessment, such as accelerometers [55]. This overestimation may be driven by social desirability bias or by the respondents’ misclassification of sedentary or light activity as moderate activity [55]. It is important to note, that in this study, the field workers who carried out data collection were all extensively trained to decrease judgmental verbal and non-verbal communication with the aim of minimizing social desirability bias. Fourth, the STEPs questionnaire was not designed to specifically test the population’s adherence to the QDGs, a fact that has limited the assessment of adherence to specific dietary recommendations. However, the availability of the WHO STEPs survey data provided a valuable opportunity to assess and generate baseline data on the lifestyle and food consumption practices of Qatari adults and their adherence to the QDGs, which can inform the development of effective and culture-specific interventions aiming at promoting adherence to the guidelines in Qatar. Lastly, the use of a value of 0 or 1 to assess a person’s adherence to the QDGs could lead to a strict classification of the individual as adherent/ not adherent to a certain guideline. While such a classification could be acceptable in epidemiological studies, a range of acceptable limit is warranted in clinical and diagnostic settings.

## Conclusions

In conclusion, this study is the first from the region to go beyond the development aspect of food-based dietary guidelines, providing data on the population’s adherence to these guidelines, its determinants and association with metabolic abnormalities. The study’s findings have characterized the gaps between the QDGs and the existing dietary and lifestyle habits of Qatari adults, identified obesity and the intakes of fruits, vegetables, legumes and whole grains as priority areas for intervention in Qatar and highlighted women, younger adults, the least educated and the unemployed as vulnerable population groups that should be targeted by specific dietary and lifestyle-based interventions. The observed association between adherence to the QDGs and decreased cardiometabolic risk serve to further validate the health benefits of the QDGs in the local context of Qatar, and call for the development of culture- specific programs and interventions aiming at promoting adherence to these guidelines.

## Appendix 1

**Table 7:** Comparing sample percentages to census percentages by gender, age and municipality

Age group (years)	Municipality (percentages)						
	Doha	Al Rayyan	Al Wakra	Umm Slal	Al khora and Al Thakhira	Madinat Al Shamal	Al Daayen
A. Comparing sample percentages to census percentages by age and municipality (Males)							
18–24	6.50	4.44	2.22	0.66	1.57	0.07	0.33
	**5.79**	**11.93**	**0.88**	**2.28**	**1.58**	**0.18**	**0.88**
25–34	17.22	8.85	6.67	1.43	5.14	0.15	0.84
	**6.32**	**12.63**	**2.98**	**2.98**	**0.70**	**0.00**	**1.75**
35–44	11.55	5.57	4.30	0.86	3.33	0.10	0.56
	**8.77**	**14.21**	**1.40**	**3.33**	**1.05**	**0.35**	**1.05**
45–54	5.94	2.88	1.97	0.41	1.48	0.05	0.27
	**5.61**	**5.09**	**0.53**	**2.11**	**0.53**	**0.00**	**0.70**
55–64	2.25	1.13	0.64	0.15	0.36	0.02	0.09
	**1.75**	**2.46**	**0.18**	**0.00**	**0.00**	**0.00**	**0.00**
B. Comparing sample percentages to census percentages by age and municipality (Females)							
18–24	7.51	6.44	1.54	1.01	0.62	0.07	0.51
	**6.31**	**11.13**	**0.74**	**4.08**	**1.30**	**0.19**	**0.37**
25–34	19.54	11.39	3.73	1.75	1.35	0.12	1.04
	**6.68**	**12.99**	**2.04**	**3.90**	**0.93**	**1.11**	**1.67**
35–44	11.48	8.75	2.38	1.55	1.08	0.10	1.09
	**7.98**	**12.99**	**1.86**	**4.27**	**0.74**	**0.19**	**1.67**
45–54	5.53	4.18	0.86	0.64	0.45	0.05	0.44
	**4.45**	**5.38**	**1.30**	**3.15**	**0.00**	**0.19**	**0.19**
55–64	2.06	1.75	0.30	0.30	0.13	0.02	0.23
	**0.74**	**1.11**	**0.19**	**0.00**	**0.00**	**0.19**	**0.00**

Bolded percentages are for the study sample distribution

## Abbreviations

BMI: body mass index
CMD: cardiometabolic disease
FAO: Food and Agriculture Organization of the United Nations
FFQ: food frequency questionnaire
GCC: Gulf Cooperation Council
GDP: Gross Domestic Product
HDL: High-density lipoprotein
MENA: Middle-East and North Africa
MetS: metabolic syndrome
NCDs: non-communicable diseases
QDGs: Qatar Dietary Guidelines
SPSS: Statistical Package for the Social Sciences
TG: triglyceride
WC: waist circumference
WHO: World Health Organization
